# Exercise breaks prevent attenuation in cerebrovascular function following an acute bout of uninterrupted sitting in healthy children

**DOI:** 10.1113/EP091314

**Published:** 2023-09-20

**Authors:** Christine M. Tallon, Daniela Nowak‐Flück, Mathew G. Reiger, Daniel J. Green, Mark S. Tremblay, Phil N. Ainslie, Ali M. McManus

**Affiliations:** ^1^ Centre for Heart, Lung, and Vascular Health, School of Health and Exercise Sciences University of British Columbia Kelowna British Columbia Canada; ^2^ School of Human Science (Sport and Exercise Sciences) The University of Western Australia Perth Western Australia Australia; ^3^ Children's Hospital of Eastern Ontario Research Institute Ottawa Ontario Canada

**Keywords:** cerebrovascular reactivity, children, sedentary

## Abstract

The purpose of this study was to examine the effect of an acute bout of prolonged sitting with and without exercise breaks on cerebrovascular function in 7‐ to 13‐year‐old children. Forty‐two children and adolescents were recruited to a crossover trial, with 15 girls (mean age 10.1 ± 2.5 years) and 16 boys (mean age 10.5 ± 1.3 years) completing the two trial conditions: SIT, uninterrupted sitting for 3 h and CYCLE, 3 h of sitting interrupted hourly with a 10‐min moderate intensity exercise break. Cerebrovascular function was measured Pre and Post SIT and CYCLE from blood flow ($\dot{Q}$), diameter, and shear rate of the internal carotid artery (ICA) at rest and in response to CO_2_. Blood velocity in the middle (MCA) and posterior (PCA) cerebral arteries was assessed at rest, during a neurovascular coupling task (NVC) and in response to CO_2_. We demonstrate that SIT but not CYCLE reduced ICA cerebrovascular reactivity to CO_2_ (%Δ ICA $\dot{Q}$/Δ end‐tidal CO_2_: SIT: Pre 5.0 ± 2.4%/mmHg to Post 3.3 ± 2.8%/mmHg vs. CYCLE: Pre 4.4 ± 2.3%/mmHg to Post 5.3 ± 3.4%/mmHg, *P* = 0.05) and slowed the MCA blood velocity onset response time to hypercapnia (SIT: Pre 57.2 ± 32.6 s to Post 76.6 ± 55.2 s, vs. CYCLE: Pre 64.1 ± 40.4 s to Post 52.3 ± 28.8 s, *P* = 0.05). There were no changes in NVC. Importantly, breaking up prolonged sitting with hourly exercise breaks prevented the reductions in cerebrovascular reactivity to CO_2_ and the slowed intracranial blood velocity onset response time to hypercapnia apparent with uninterrupted sitting in children.

## Abstract (highlights)

- **What is the central question of this study?**
  What are the effects of interrupting prolonged sitting on cerebrovascular function in children?
- **What is the main finding and its importance?**
  Prolonged sitting results in declines in cerebrovascular reactivity, a valuable index of cerebrovascular health. Breaking up prolonged sitting with hourly 10 min exercise breaks prevented these changes. These initial findings suggest excessive sedentary behaviour does impact cerebrovascular function in childhood, but taking exercise breaks prevents declines.

## 1 INTRODUCTION

Childhood sedentary behaviour was already at alarming levels before the COVID‐19 pandemic (Saunders, 2014); however, as children have transitioned to a largely seated digital environment for schooling and social interaction during country‐wide lockdowns and restrictions, sedentary time increased by an average of 160 min per day (Runacres et al., 2021). The health impacts of such increases in sedentary behaviour in childhood have yet to be fully elucidated. This is worrisome, given evidence that excessive sedentary time is an independent risk factor for cardiovascular disease in both children and adults (Hamilton et al., 2008; Saunders et al., 2014).

The muscular inactivity that accompanies excessive sedentary time, time largely spent seated, results in well‐documented deleterious outcomes, including detrimental shifts in metabolic processes, particularly glucose metabolism, and alterations in cardiovascular function (Hamilton, 2018). In various models of enforced sitting, evidence indicates that acute bouts of sitting contribute to peripheral vascular deconditioning in adults and children (McManus et al., 2015; Taylor et al., 2022; Thosar et al., 2015). In adults these reductions are related to reduced shear stress, which decreases the availability of nitric oxide and increases the production of vasoconstrictors (Thosar et al., 2012), suggesting sitting alters endothelial integrity (Thosar et al., 2015). Far less is understood about the cerebrovascular changes that accompany bouts of prolonged sitting; however, reductions in middle cerebral artery (MCA) blood velocity of 2.5–5.5% have been documented in adults following an acute bout of enforced sitting (Carter et al., 2018; Wheeler et al., 2019). Importantly, these declines were prevented by taking exercise breaks, even short‐duration breaks of 2–3 min every 30 min (Carter et al., 2018). Although no study, to the best of our knowledge, has examined the impact of a bout of prolonged sitting on cerebral blood flow or cerebrovascular function in children, there is evidence that a lower internal carotid artery (ICA) shear rate is associated with high levels (>452 min of per day) of sedentary behaviour (Tallon et al., 2021) in both boys and girls. This level of sedentary behaviour has become common among some populations during COVID (Runacres et al., 2021), and determining the impact of prolonged sitting on cerebrovascular vasodilatory function in children should be a priority.

In contrast to excessive sitting, light to moderate‐intensity exercise activates metabolic pathways that appear to positively impact brain perfusion (Ellis et al., 2017; Tallon et al., 2019) and cognitive function in children (Hillman et al., 2015). In adults, there is evidence that breaking up sitting with light‐intensity exercise improves cognitive function (Mullane et al., 2017; Wheeler et al., 2020); however, an exercise‐induced increase in cognitive function has not been found to relate to alterations in brain blood flow in children (Pontifex et al., 2018). More sensitive markers of cerebrovascular function may be needed when examining relationships among excessive sitting, sedentary behaviour and exercise. The pattern of the cerebrovascular onset response to a given stimuli such as CO_2_ or exercise can be derived from kinetic modelling. The commonly used single exponential model with delay term describes this response, providing valuable information on the speed (i.e., delay term, tau (τ), and mean response time (MRT)), as well as the magnitude or amplitude (Δ_A_) of the response (Billinger et al., 2017; Kempf et al., 2019; Ogoh et al., 2009; Poulin et al., 1996). This approach has provided additional and complementary insight into developmental differences in the regulation of cerebral blood flow. A markedly slower τ in response to hypercapnia was shown for MCA blood velocity (Tallon et al., 2020) and ICA flow (${\dot{Q}}_{\mathrm{ICA}}$; Tallon et al., 2022) in children compared to adults, supporting an age‐dependent response to hypercapnia in the extra‐ and intra‐cranial vessels. Measuring changes in not only the magnitude of the response but also the speed may be important for determining the impact of prolonged sitting on cerebrovascular function, as well as any protective effect breaking up sitting with exercise may impart.

Taken together, the extant literature would suggest prolonged sitting reduces cerebral blood flow. Therefore, the purpose of this study was to examine the effect of an acute bout of prolonged sitting on cerebral blood flow and function in children, and to determine whether exercise breaks afford protection against sitting‐induced reductions in cerebral blood flow or cerebrovascular function. We hypothesized that, controlling for maturation, 3 h of uninterrupted sitting in 7‐ to 13‐year‐old children would result in decreased cerebrovascular function (assessed from neurovascular coupling (NVC) and cerebrovascular reactivity to CO_2_), whereas 3 h of sitting interrupted by hourly 10‐min exercise breaks will prevent these declines.

## 2 METHODS

### 2.1 Ethical approval

This study was approved by the clinical research ethics board of the University of British Columbia (H16‐01324). All experimental protocols and procedures conformed to the standards set by the Canadian government Tri‐Council policy statement for integrity in research, as well as the *Declaration of Helsinki*, except for registration in a database. A detailed verbal and written explanation of the procedures and measurements was provided to participants and parents/guardians before participation. Written informed consent was obtained from the parents/guardians of participants, alongside written and oral informed assent from participants.

### 2.2 Participants

Forty‐two children and adolescents (7–13 years of age) were recruited from the local community and volunteered to participate in this study. Individuals were excluded if they were unable to exercise on a stationary cycle ergometer, had known congenital abnormalities or respiratory disease, known high blood pressure, dyslipidaemia or high insulin. None of the children were taking medications or supplements.

### 2.3 Experimental design

**FIGURE 1:**
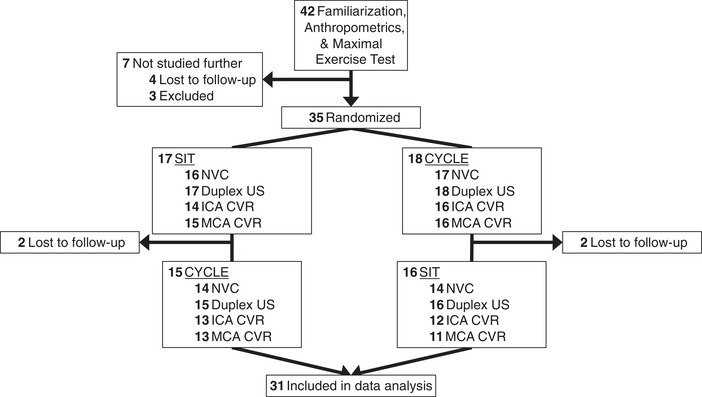
Progression of participants from the point of recruitment until completion of the experimental trial. CVR, cerebrovascular reactivity to CO_2_; ICA, internal carotid artery; MCA, middle cerebral artery; NVC, neurovascular coupling; US, ultrasound.

A crossover experimental design was utilized with two experimental conditions: (i) uninterrupted sitting (SIT) and (ii) breaks in sitting time (CYCLE). The two conditions were completed on two separate days less than 28 days apart, the order of which was randomized. Figure 1 depicts the progress of participants from recruitment to completion of the trial.

Participants visited the laboratory, at the same time of day, on three separate occasions. They were asked to report to the laboratory 2 h after eating the same light, low‐fat meal at a scheduled time (between 08.00 and 10.00 h) and asked to refrain from caffeine, alcohol or strenuous exercise 24 h before testing.

The first visit consisted of familiarization with the laboratory and the protocols involved in the experimental conditions. Participants completed a baseline assessment of body composition and anthropometrics, followed by an exercise test on a cycle ergometer used to determine the standardized intensity of the exercise breaks. Participants were instrumented with a thigh‐worn accelerometer at the end of this visit and wore the device for 7 days prior to returning to the laboratory for the experimental conditions. Participants returned to the laboratory on two subsequent occasions, with a minimum of 24 h between each visit, to complete the two experimental conditions.

The two experimental conditions consisted of:

- SIT: supervised uninterrupted sitting. Participants sat for 3 h with their feet on the ground and knees bent at 90°. If a bathroom break was required, participants were pushed in an office chair on wheels to the bathroom to minimize movement.
- CYCLE: supervised interrupted sitting. Participants also sat for 3 h, but sitting was interrupted with a 10 min cycle ergometer exercise break each hour, cycling at an individualized moderate intensity (90% of ventilatory threshold, determined from the familiarization session exercise test). Three cycling breaks were completed at minutes 25–35, 85–95 and 145–155. When not cycling, participants returned to sitting under the same guidelines as followed in the SIT condition noted above.

**FIGURE 2:**
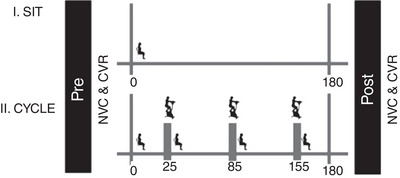
Illustration of experimental design of the two conditions. CVR, cerebrovascular reactivity to CO_2_; NVC, neurovascular coupling.

Assessment of cerebrovascular function was completed before (Pre) and following (Post) both the SIT and CYCLE experimental conditions. Figure 2 illustrates the experimental design and the timing of the exercise breaks.

Following the Pre assessments, participants were taken to a mock sitting room, with comfortable seating and access to colouring books and iPads with a choice of parental approved apps. These sources of entertainment were available to participants during both conditions to increase the ecological validity of the study by replicating the home environment. The children largely played on game type apps on the iPads, such as Fruit Ninja, Candy Crush, Cut the Rope and Temple Run. Some of the children chose to watch a movie on the Netflix Kids app and two of the boys brought their own PlayStation to play their own sport game. None used the colouring books or read, and only two children used an educational app, playing a math game app.

Once seated, Pre circumference of the right calf was recorded as an approximation of venous pooling that may have occurred during the sustained seated posture. For the duration of the two experimental conditions participants wore an activPAL micro‐accelerometer (PAL Technologies Ltd; Glasgow, UK) to quantify total sitting time and total cycling time. Following the attachment of the micro‐accelerometer to the anterior mid‐thigh, the 3 h experimental condition commenced. Upon completion of the 3 h experimental condition, Post calf circumference was recorded and participants were wheeled in an office chair into the laboratory where Post assessment of cerebrovascular function was completed.

### 2.4 Measures

#### 2.4.1 Body composition

Assessment of body mass, stature and sitting height was completed with participants barefoot and in light clothing. Body mass was recorded to the nearest 0.1 kg (Tanita electronic scale; Tanita Corp, Arlington Heights, IL, USA) and both stature and sitting height were recorded to the nearest 0.1 cm (Seca Portable Stadiometer; Seca, Hamburg, Germany). Right calf circumferences were measured to the nearest 0.1 cm, using an anthropometric tape, following the International Society for the Advancement of Kinanthropometry protocol (Norton & Olds, 1996). Body composition (fat mass and fat‐free mass) was assessed from a whole‐body dual‐energy X‐ray absorptiometry (DXA) scan (Horizon DXA System, Hologic Canada ULC, Mississauga, Canada).

#### 2.4.2 Maturation

Maturation was assessed from predicted age at peak height velocity (aPHV) using the Mirwald equation (Mirwald et al., 2002), which provides estimates of maturity offset from somatic measures. A negative offset value (⩽−1.0) indicates the participant has yet to reach aPHV (pre‐PHV), a score between −1.0 and 1.0 indicates the participant is within the year that aPHV occurs (peri‐PHV) and a positive offset value (≥1.0) indicates the participant has already passed aPHV (post‐PHV).

#### 2.4.3 Assessment of sitting and cycling time during the experiment, as well as habitual physical activity and sedentary behaviour

Total sitting and cycling time during the experimental conditions, as well as habitual physical activity and sedentary behaviour, was recorded using the activPAL micro‐accelerometer (PAL Technologies). Participants wore the device on the anterior aspect of the right thigh, placed within a flexible nitrile sleeve and attached using Tegaderm Transparent Film (3M Health Care; St Paul, MN, USA). Leg‐worn devices are the gold standard for the assessment of sedentary behaviour, and the activPAL has been shown to provide validated measures for sedentary behaviour, step‐based activity and posture in children (Aminian & Hinckson, 2012; Ridley et al., 2016). The activPAL micro‐accelerometer was set at a sampling frequency of 20 Hz and data were interpolated in 2 s bins using PALanalysis software (version 8, PAL Technologies). The accelerometry data recorded during the experimental conditions were analysed for total sitting time and total time cycling. For habitual physical activity and sedentary behaviour participants were asked to wear the device continuously for seven consecutive days. ActivPAL data inclusion criteria included: (i) 24 h of wear time per day, and (ii) ≥3 weekdays and ≥1 weekend day. Of the 31 who completed the trial, 22 met these criteria. Waking time was defined as the duration of time between the first steps or standing in the morning and the cessation of steps or standing in the evening. Daily step count averages and total sedentary time (calculated from total time spent sitting or lying during the waking day) were calculated using a 7‐day weighted (5:2 weekday to weekend day) average and inflating individual data that were less than 5 weekdays or 2 weekend days using the individual means.

#### 2.4.4 Exercise break intensity

The power (W) at 90% of ventilatory threshold was used as the intensity for the CYCLE condition. Ventilatory threshold was identified using a ramp exercise test on a child‐sized electromagnetically braked cycle ergometer (Lode Pediatric Corival, Groningen, Netherlands). Following a 3‐min unloaded warm‐up, the increments were height dependent: 10 W increments for participants 110–125 cm in stature; or 15 W increments for participants 125–150 cm in stature. Participants were asked to maintain a cadence of 70–80 revolutions per minute until volitional exhaustion, determined as a drop of cadence of >10 revolutions per minute despite encouragement. Ventilatory threshold was confirmed manually using the V‐slope method (Beaver et al., 1986).

#### 2.4.5 Cardiovascular measures

Heart rate and blood pressure were measured continuously during Pre and Post assessments. Heart rate was assessed using a three‐lead electrocardiogram (ADInstruments BioAmp ML132, Colorado Springs, CO, USA), while blood pressure was assessed using a Finometer Pro (Finapres Medical Systems, Enschede, Netherlands). All variables were sampled continuously at 1 kHz via an analog‐to‐digital converter (Powerlab, 16.30, ADInstruments) and down‐sampled for processing at 1 Hz. Mean arterial pressure was calculated as diastolic pressure plus one‐third of systolic blood pressure minus diastolic blood pressure.

#### 2.4.6 Cerebrovascular measures

The following describes the cerebrovascular assessments undertaken Pre and Post experimental conditions. The order presented below matches the order in which participants completed these measures. Data processing was completed blinded to the time point and condition.

The NVC test was used to assess the temporal and spatial coupling of regional intra‐cranial blood velocity and metabolic demand (Phillips et al., 2016). Intra‐cranial blood velocity of the left posterior cerebral artery (PCA) and right MCA was assessed using a 2 MHz transcranial Doppler (TCD) ultrasound (Spencer Technologies, Redmond, WA, USA). Two TCD probes were attached to a headpiece (child‐adapted M600 bilateral head frame; Spencer Technologies) and secured in order to insonate the blood vessels through the trans‐temporal window following established guidelines (Willie et al., 2011). Activation of the visual cortex was used to evaluate changes to the PCA velocity, in comparison to an assessment of regional MCA velocity. The change in PCA velocity and MCA velocity from baseline in response to activation of the visual cortex was recorded over five cycles of repeating and alternating 30 s of exposure to eyes‐closed, and 30 s of an eyes‐open visual searching task. The visual searching task involved looking for Where's Waldo (Wally) characters (Smirl et al., 2016) and was presented via a Microsoft PowerPoint presentation, automatically switching between a black screen and a random Where's Waldo illustration. In order to standardize the transition, an auditory cue was used at the transition point between eyes‐open and eyes‐closed to cue the children. The PCA velocity and MCA velocity response to the five cycles were averaged and analysed using custom software developed in MATLAB (The MathWorks, Natick, MA, USA; Phillips et al., 2016). A 40 s average before the start NVC test was used as baseline and a 25 s average during the eyes‐closed/eyes‐open cycles were used to calculate percentage change from baseline.

The hypercapnic cerebrovascular reactivity protocol utilized in this study has previously been described (Tallon et al., 2020, 2022). In brief, following a 10 min period of resting supine, participants breathed room air for 2 min followed by 4 min of breathing a fixed concentration of 6% inspired CO_2_, administered in 21% O_2_, remainder N_2_. A three‐way Hans Rudolph (Shawnee, KS, USA) valve was used to switch between room air and a 20 litre Douglas bag containing the 0.06 $F_{{\mathrm{ICO}}_{2}}$. End‐tidal carbon dioxide ($P_{\mathrm{ETC}O_{2}}$) and oxygen ($P_{\mathrm{ET}O_{2}}$) and ventilation ($\dot{V_{E}}$) were assessed on a breath‐by‐breath basis using a gas analyser (ML206, ADInstruments) connected to an analog‐to‐digital converter (Powerlab 16/30, ADInstruments). This allowed for continuous breath‐by‐breath sampling of end‐tidal carbon dioxide ($P_{\mathrm{ETC}O_{2}}$) and the end‐tidal oxygen ($P_{\mathrm{ET}O_{2}}$). These measures were time‐aligned with the cerebrovascular measures, also connected to the analog‐to‐digital converter. The pneumotach (HR800L, Hans Rudolph) was calibrated prior to every test using a 3‐litre syringe, and the gas analysers were calibrated using gases of known concentration.

Cerebrovascular measures included intra‐cranial blood velocity of the MCA and PCA as described above and volumetric assessment of the ICA. A 15 MHz multi‐frequency array vascular ultrasound probe (Terason T3200, Teratech, Burlington, MA, USA) was used unilaterally (right side) for volumetric assessment of ICA blood velocity and vessel diameter. B‐mode imaging was used to measure arterial diameter, while pulse‐wave mode was used to simultaneously measure peak blood velocity. Recordings were screen‐captured and stored as video files for offline analysis (Woodman et al., 2001). Technical recommendations were followed for all ICA measures (Thomas et al., 2015), including visual inspection before analysis. Exclusion of recordings was based on three criteria: (i) the occurrence of an overt angle change, (ii) excessive movement of the vessel as a result of high $\dot{V_{E}}$, and (iii) overall poor image quality (e.g., unclear vessel walls).

Synchronized diameter and velocity recordings allowed the calculation of ${\dot{Q}}_{\mathrm{ICA}}$ and shear rate (ICA_Shear‐rate_). Volumetric blood flow was calculated as:

$$\dot{Q}\;\left(\mathrm{mL}\mathrm{mi}n^{-1}\right)\;=\;\frac{\mathrm{peak}\;\mathrm{envelope}\;\mathrm{velocity}}{2}\times \left[\pi {\left(0.5\times \mathrm{diameter}\right)}^{2}\right]$$

Shear rate was calculated as:

$$\mathrm{Shear}\;\mathrm{rate}\;\left(s^{-1}\right)\;=\;\left(\frac{4\times \mathrm{velocity}}{\mathrm{diameter}}\right)$$

Baseline values were calculated from the 2 min of supine rest and response values were averaged across the final 30 s of the 4‐min hypercapnic test.

Steady state cerebrovascular reactivity to CO_2_ of the MCA velocity, PCA velocity and ${\dot{Q}}_{\mathrm{ICA}}$ to the hypercapnic stimulus were computed as previously described (Tallon et al., 2020, 2022). Absolute cerebrovascular reactivity to CO_2_ was calculated as:


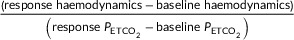




Relative cerebrovascular reactivity to CO_2_ as:


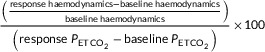




The dynamic intra‐ and extra‐cranial onset response to hypercapnia was calculated as previously described (Tallon et al., 2020, 2022). In brief, the ICA haemodynamic bins (1 Hz) were passed through a median filter (with a rank of 7). A mono‐exponential model with a delay term was used to explore the onset response of MCA velocity, PCA velocity, ${\dot{Q}}_{\mathrm{ICA}}$ and ICA ICA_Shear‐rate_, to hypercapnia (GraphPad Prism v.9.0.1; GraphPad Software, San Diego, CA, USA):

$$y\left(t\right)=y_{0}+\;\Delta_{A}\;\left(1-e-\frac{t-\mathrm{TD}}{\tau }\right)$$

where y(*t*), y_0_, Δ_A_, TD and τ are the response at a given time, the baseline value, the baseline corrected change in amplitude from baseline to asymptote, the time delay and the time constant of the response, respectively.

The response to hypercapnia of each participant was modelled from the onset of the 6% CO_2_ stimulus (0 s). Outliers within each participant's modelled response were detected and removed to optimize fit of the mono‐exponential model using the robust regression and outlier removal method within the Prism software (Motulsky & Brown, 2006). Goodness of fit (*r* ^2^ > 0.50) and normality of residuals were used to determine model acceptability. The mean response time was calculated for ${\dot{Q}}_{\mathrm{ICA}}$, as: Mean response time = TD + τ.

### 2.5 Statistical analyses

Statistical analyses were performed using SPSS 28.0 (SPSS, IBM Corp., Armonk, NY, USA). All data are reported as means ± standard deviation (SD). Normality was checked and verified using the Shapiro–Wilk normality test for variables at baseline. Student's *t*‐test was used to compare baseline characteristics between boys and girls. Repeated measures analyses of variance (RM‐ANOVA) were used to examine the main effects of time (Pre and Post) and condition (SIT and CYCLE), as well as interactions for the primary outcome variables. Maturation (aPHV) was included as a covariate in all RM‐ANOVA analyses. Where necessary, main effects and interactions were deconstructed using paired samples *t*‐tests with Bonferroni corrections. Pearson correlations between steps per day, sedentary time, resting MCA velocity, ${\dot{Q}}_{\mathrm{ICA}}$ and ICA_Shear‐rate_, as well as absolute and relative MCA velocity and ${\dot{Q}}_{\mathrm{ICA}}$ reactivity to CO_2_ and the kinetic onset responses for MCA velocity and ${\dot{Q}}_{\mathrm{ICA}}$ were computed. Alpha was set at *P* ≤ 0.05.

## 3 RESULTS

As noted in Figure 1, of the 35 participants randomized (19 girls and 16 boys), 31 (age range: 7.8–13.1 years; 15 girls) completed the experimental conditions. Table 1 presents baseline characteristics of the initial 35 participants randomized, as well as the 31 who completed. The four participants who dropped out were all girls and were significantly leaner than the remaining 31 who completed the trial. Of the 31 completers, boys were less mature than girls as indicated by the difference in predicted aPHV offset score (*P* *=* 0.004). None of the girls were menarchal. Boys had a lower body fat than the girls (*P* = 0.003), but none of the participants were overweight or obese.

**TABLE 1:** Participants’ demographic and anthropometric characteristics.

	All (*n* = 35)	Completers (*n* = 31)	Girls (*n* = 15)	Boys (*n* = 16)
Age (years)	10.2 ± 1.4	10.3 ± 1.4	10.1 ± 1.5	10.5 ± 1.3
Stature (cm)	145.1 ± 12.1	146.4 ± 11.7	143.6 ± 10.9	149.0 ± 12.1
Sitting height (cm)	73.2 ± 5.5	73.6 ± 5.6	73.6 ± 5.9	73.7 ± 5.6
Mass (kg)	40.1 ± 13.5	41.4 ± 13.6	42.3 ± 14.8	40.6 ± 12.7
Body mass index (m kg^−2^)	18.6 ± 4.0	18.9 ± 4.13	20.0 ± 4.4	18.0 ± 3.7
aPHV (years offset)	−2.1 ± 1.5	−2.1 ± 1.5	−1.3 ± 1.4	−2.8 ± 1.2^*^
Waist circumference^a^ (cm)	65.6 ± 9.1	65.6 ± 9.1	64.2 ± 7.2	66.7 ± 10.5
Fat mass (kg)	10.2 ± 6.6	10.2 ± 6.6	11.9 ± 6.8	8.5 ± 6.1
Lean mass (kg)	29.4 ± 8.3	29.4 ± 8.3	28.6 ± 8.3	30.1 ± 7.8
Body fat (%)	23.3 ± 7.2	23.3 ± 7.2	27.1 ± 5.2	19.8 ± 7.1^*^

*Note*: Values are means ± SD unless otherwise specified.

^a^ Waist circumference was not recorded for two girls (*n* = 13).

* *P* < 0.05.

Abbreviation: aPHV, age at predicted peak height velocity.

During the CYCLE condition participants cycled at a mean intensity of 41.5 ± 13.6 W, and engaged in significantly less time sitting (146.9 ± 6.3 vs. 178.4 ± 4.3 min, *P* < 0.001). There was no main effect for time (*P* = 0.643) or condition (*P* = 0.977) for calf circumference (Pre CYCLE 30.2 ± 4.5 cm, Post CYCLE 30.4 ± 4.4 cm vs. Pre SIT 30.2 ± 4.5 cm, Post SIT 30.5 ± 4.5 cm).

### 3.1 Neurovascular coupling

Of the 31 children who completed the experimental conditions, 12 did not complete the Pre or Post NVC test. Of the 12, PCA could not be insonated in eight. The remaining four did not complete the NVC test properly (e.g., opened eyes during closing, or did not search for Waldo/Wally). Therefore, data from the NVC test are presented on 19 participants (eight girls). Baseline and peak response did not differ significantly across time or by condition for any variable (Table 2). When accounting for baseline and assessing the change in both absolute and percentage terms, there was no significant difference across time or by condition for any variable.

**TABLE 2:** Cardiovascular and cerebral neurovascular coupling parameters pre and post both experimental conditions.

							*P* (ANOVA main effects)		
	*n*		Pre SIT	Post SIT	Pre CYCLE	Post CYCLE	Time	Condition	Time × Condition
MAP (mmHg) (8 girls)	19	BL	70 ± 17	66 ± 20	66 ± 18	66 ± 16	0.401	0.643	0.488
		Peak	75 ± 19	71 ± 19	70 ± 19	71 ± 17	0.474	0.653	0.407
		ΔAbs	5 ± 3	5 ± 4	4 ± 3	5 ± 5	0.566	0.956	0.508
		Δ%	7.4 ± 3.8	10.1 ± 11.0	7.7 ± 7.2	9.7 ± 11.4	0.117	0.990	0.841
HR (bpm) (8 girls)	19	BL	85 ± 11	81 ± 11	84 ± 9	83 ± 10	0.506	0.912	0.267
		Peak	92 ± 8	88 ± 11	91 ± 8	92 ± 12	0.848	0.602	0.136
		ΔAbs	7 ± 5	7 ± 4	7 ± 6	9 ± 8	0.708	0.389	0.490
		Δ%	8.7 ± 6.1	9.7 ± 5.2	8.9 ± 7.5	12.1 ± 10.8	0.685	0.503	0.492
PCA velocity (cm s^−1^) (8 girls)	19	BL	41.4 ± 10.9	40.8 ± 10.0	41.3 ± 10.4	40.9 ± 10.9	0.969	0.995	0.929
		Peak	52.5 ± 12.9	52.6 ± 12.3	52.7 ± 12.8	51.6 ± 13.8	0.610	0.922	0.675
		ΔAbs	11.1 ± 4.2	11.8 ± 3.5	11.4 ± 3.7	10.8 ± 4.5	0.273	0.740	0.316
		Δ%	28.1 ± 10.3	29.9 ± 8.8	28.3 ± 8.6	27.2 ± 9.3	0.685	0.599	0.449
PCACVC (cm s^−1^ mmHg^−1^)	19	BL	0.65 ± 0.32	0.71 ± 0.36	0.69 ± 0.27	0.68 ± 0.31	0.174	0.976	0.598
		Peak	0.88 ± 0.52	0.94 ± 0.39	0.93 ± 0.46	0.86 ± 0.33	0.372	0.887	0.388
		ΔAbs	0.23 ± 0.21	0.23 ± 0.12	0.25 ± 0.21	0.17 ± 0.14	0.670	0.689	0.278
		Δ%	34.7 ± 17.1	36.1 ± 16.1	33.9 ± 16.4	29.3 ± 14.1	0.740	0.351	0.358
MCA velocity (cm s^−1^) (8 girls)	19	BL	70.6 ± 7.4	69.4 ± 8.9	67.4 ± 18.0	70.9 ± 9.0	0.723	0.786	0.287
		Peak	78.4 ± 10.8	78.0 ± 10.0	75.2 ± 20.2	77.3 ± 9.9	0.594	0.596	0.616
		ΔAbs	7.8 ± 5.1	8.6 ± 3.6	7.8 ± 5.2	6.4 ± 3.7	0.071	0.155	0.353
		Δ%	11.2 ± 6.7	13.0 ± 5.8	11.4 ± 8.1	9.7 ± 6.1	0.089	0.159	0.396
MCACVC (cm s^−1^ mmHg^−1^) (8 girls)	19	BL	1.12 ± 0.46	1.24 ± 0.67	1.14 ± 0.56	1.17 ± 0.36	0.212	0.888	0.679
		Peak	1.31 ± 0.69	1.44 ± 0.71	1.32 ± 0.68	1.30 ± 0.40	0.334	0.697	0.559
		ΔAbs	0.19 ± 0.24	0.20 ± 0.16	0.18 ± 0.21	0.13 ± 0.20	0.808	0.387	0.470
		Δ%	15.8 ± 13.3	19.0 ± 12.9	15.2 ± 13.5	12.5 ± 10.8	0.709	0.266	0.243

*Note*: Values are means ± SD. Abbreviations: BL, baseline; ΔAbs, absolute change from baseline value to peak value; Δ%, change to peak relative to baseline value; HR, heart rate; MAP, mean arterial pressure; MCA, middle cerebral artery; MCACVC, middle cerebral artery cerebrovascular conductance index; PCA, posterior cerebral artery; PCACVC, posterior cerebral artery cerebrovascular conductance index.

### 3.2 Baseline and steady‐state responses to hypercapnia

Absolute values for all variables at baseline and during the last 30 s of the 4 min hypercapnic challenge are presented in Table 3. Nine participants refused either the Pre or Post hypercapnic challenge. Of the 22 participants who did complete the hypercapnic challenge at all four time points, two participants were excluded due to baseline data exceeding resting values (i.e., heart rate > 140 bpm, $P_{\mathrm{ET}O_{2}}$ > 130 mmHg, or $P_{\mathrm{ETC}O_{2}}$ < 25 mmHg) at three or more of the assessments. Additionally, of the 20 participants (eight girls) included in the analysis, not all participants had MCA, PCA and ICA data collected, and therefore the *n* for each vessel is presented in Table 3. Baseline heart rate, mean arterial pressure, $P_{\mathrm{ET}O_{2}}$, $P_{\mathrm{ETC}O_{2}}$, MCA velocity, PCA velocity, ICA_Shear‐rate_ or ICA_Diameter_ revealed no significant effect of time, condition or interaction at baseline. A significant time by condition interaction was identified for baseline ICA_Velocity_ (*P* = 0.029) and ${\dot{Q}}_{\mathrm{ICA}}$ (*P* = 0.011), but not for hypercapnia.

**TABLE 3:** Cardiovascular and cerebrovascular parameters pre‐ and post‐hypercapnia.

							*P* (ANOVA main effects)		
	*n*		Pre SIT	Post SIT	Pre CYCLE	Post CYCLE	Time	Condition	Time × Condition
HR (beats min^−1^) (8 girls)	20	BL	78 ± 10	76 ± 12	78 ± 13	78 ± 11	0.305	0.764	0.658
		HC	91 ± 10	90 ± 13	90 ± 13	92 ± 13	0.977	0.883	0.307
		Δ	13 ± 5	13 ± 4	12 ± 6	14 ± 5	0.272	0.627	0.411
MAP (mmHg) (8 girls)	20	BL	75 ± 10	73 ± 10	75 ± 13	76 ± 12	0.513	0.643	0.399
		HC	80 ± 10	78 ± 11	80 ± 12	80 ± 12	0.827	0.820	0.567
		Δ	5 ± 9	5 ± 4	5 ± 5	4 ± 7	0.108	0.379	0.909
$P_{\mathrm{ETC}O_{2}}$ (mmHg) (8 girls)	20	BL	44.1 ± 4.5	44.1 ± 4.7	44.6 ± 5.9	44.4 ± 4.9	0.170	0.807	0.651
		HC	52.9 ± 4.3	52.8 ± 4.1	53.5 ± 5.2	52.8 ± 4.6	0.230	0.864	0.375
		Δ	8.9 ± 1.5	8.7 ± 2.3	8.8 ± 2.7	8.4 ± 1.9	0.828	0.756	0.724
$P_{\mathrm{ET}O_{2}}$ (mmHg) (8 girls)	20	BL	102.1 ± 4.4	98.4 ± 4.6	100.1 ± 5.8	98.4 ± 4.9	0.450	0.483	0.161
		HC	138.6 ± 6.4	138.5 ± 5.8	138.2 ± 6.1	138.8 ± 5.9	0.682	0.958	0.418
		Δ	36.6 ± 4.4	40.2 ± 4.7	38.5 ± 5.7	40.4 ± 6.0	0.458	0.460	0.272
MCA velocity (cm s^−1^) (6 girls)	19	BL	84.7 ± 14.5	83.7 ± 12.1	83.7 ± 15.5	84.7 ± 13.4	0.517	0.995	0.622
		HC	110.9 ± 17.3	109.1 ± 15.0	109.8 ± 20.4	108.2 ± 20.0	0.798	0.852	0.966
		Δ	26.2 ± 13.0	25.4 ± 12.6	26.1 ± 14.0	23.6 ± 16.8	0.466	0.791	0.770
PCA velocity (cm s^−1^) (5 girls)	15	BL	51.6 ± 15.7	52.3 ± 16.5	49.9 ± 15.5	51.0 ± 16.3	0.514	0.781	0.922
		HC	67.1 ± 21.1	70.6 ± 28.5	62.6 ± 20.3	63.5 ± 21.5	0.353	0.465	0.630
		Δ	15.5 ± 9.6	18.3 ± 14.1	12.7 ± 6.7	12.4 ± 7.3	0.336	0.195	0.308
ICA velocity (cm s^−1^) (2 girls)	12	BL	**57.1 ± 7.0**	61.0 ± 8.5*	**59.1 ± 8.6**	**56.7 ± 10.3**	0.444	0.743	**0.029**
		HC	78.5 ± 9.0	78.5 ± 8.7	79.5 ± 16.8	78.2 ± 8.9	0.512	0.931	0.781
		Δ	21.4 ± 5.2	17.5 ± 7.8	20.4 ± 13.0	21.2 ± 7.2	0.831	0.617	0.269
ICA_Shear‐rate_ (s^−1^) (2 girls)	12	BL	467.8 ± 73.5	485.4 ± 65.2	482.0 ± 92.7	469.5 ± 101.0	0.853	0.978	0.325
		HC	626.6 ± 109.1	618.3 ± 113.7	626.3 ± 159.6	616.1 ± 89.8	0.713	0.976	0.968
		Δ	158.8 ± 49.8	132.9 ± 68.4	144.3 ± 107.6	146.6 ± 58.8	0.747	0.988	0.440
ICA_diameter_ (mm) (2 girls)	12	BL	4.93 ± 0.40	5.02 ± 0.39	4.95 ± 0.41	4.95 ± 0.37	0.532	0.900	0.498
		HC	5.08 ± 0.41	5.10 ± 0.48	5.16 ± 0.39	5.12 ± 0.41	0.460	0.725	0.633
		Δ	0.16 ± 0.18	0.08 ± 0.28	0.22 ± 0.20	0.16 ± 0.14	0.744	0.355	0.827
${\dot{Q}}_{\mathrm{ICA}}$ (ml min^−1^) (2 girls)	12	BL	**240.5 ± 53.3**	267.9 ± 64.6*	**253.6 ± 47.8**	**234.2 ± 46.1**	0.610	0.613	**0.011**
		HC	337.1 ± 59.7	336.7 ± 82.1	353.4 ± 70.9	333.9 ± 71.8	0.720	0.790	0.523
		Δ	96.6 ± 36.2	68.8 ± 64.5	99.9 ± 49.5	99.7 ± 48.9	0.927	0.346	0.243

*Note*: Values are means ± SD. Bold indicates a significant main effect or interaction.

^*^Indicates a Condition specific Pre to Post difference (*P* < 0.05). Abbreviations: BL, baseline; HC, hypercapnia; HR, heart rate; ICA, internal carotid artery; MAP, mean arterial blood pressure; MCA, middle cerebral artery; $P_{\mathrm{ETC}O_{2}}$, end‐tidal carbon dioxide; $P_{\mathrm{ET}O_{2}}$, end‐tidal oxygen; PCA, posterior cerebral artery; ${\dot{Q}}_{\mathrm{ICA}}$, internal carotid artery blood flow.

**FIGURE 3:**
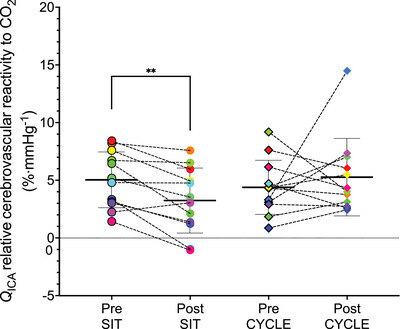
Relative cerebrovascular reactivity of the internal carotid artery Pre and Post experimental conditions. Circles represent the SIT condition and diamonds represent the CYCLE condition. The relative ${\dot{Q}}_{\mathrm{ICA}}$ cerebrovascular reactivity CO_2_ of each participant is represented by a single colour at all assessments. **P = 0.001.

In both absolute and relative terms, there was no significant main effect of time or conditions on the cerebrovascular reactivity to CO_2_ of the MCA velocity or PCA velocity (Table 4). There was a significant time by condition interaction of ${\dot{Q}}_{\mathrm{ICA}}$ relative cerebrovascular reactivity to CO_2_ (*P* = 0.05). The time by condition interaction persisted when the outlier in the CYCLE condition was removed (*P* = 0.05). Follow‐up tests indicated a lower ${\dot{Q}}_{\mathrm{ICA}}$ relative cerebrovascular reactivity to CO_2_ following SIT (CI: 0.8%–2.8%⋅mmHg^−1^, *P* = 0.001; see Figure 3), with no change in ${\dot{Q}}_{\mathrm{ICA}}$ relative cerebrovascular reactivity to CO_2_ following CYCLE (CI:−3.8% to 1.1%⋅mmHg^−1^, *P* = 0.235; see Figure 3).

**TABLE 4:** Absolute and relative cerebrovascular reactivity pre and post experimental conditions.

							*P* (ANOVA main effects)		
		*n*	Pre SIT	Post SIT	Pre CYCLE	Post CYCLE	Time	Condition	Time × Condition
MCA velocity	Cerebrovascular reactivity to CO_2_^Abs^ (cm⋅s^−1^⋅mmHg^−1^)	19	2.9 ± 1.2	2.9 ± 1.0	3.1 ± 1.6	2.9 ± 2.1	0.515	0.817	0.867
	Cerebrovascular reactivity to CO_2_^Rel^ (%⋅mmHg^−1^)	19	3.6 ± 1.5	3.5 ± 1.3	3.8 ± 2.5	3.5 ± 2.7	0.356	0.817	0.792
PCA velocity	Cerebrovascular reactivity to CO_2_^Abs^ (cm⋅s^−1^⋅mmHg^−1^)	15	1.8 ± 1.0	2.0 ± 1.3	1.6 ± 1.1	1.5 ± .9	0.457	0.346	0.370
	Cerebrovascular reactivity to CO_2_^Rel^ (%⋅mmHg^−1^)	15	3.2 ± 1.3	3.7 ± 1.6	3.2 ± 1.6	3.0 ± 1.6	0.798	0.458	0.441
${\dot{Q}}_{\mathrm{ICA}}$	Cerebrovascular reactivity to CO_2_^Abs^ (cm⋅s^−1^⋅mmHg^−1^)	12	11.5 ± 4.9	8.3 ± 7.1	11.1 ± 6.1	12.0 ± 6.7	0.916	0.419	0.184
	Cerebrovascular reactivity to CO_2_^Rel^ (%⋅mmHg^−1^)	12	**5.0 ± 2.4**	3.3 ± 2.8*	**4.4 ± 2.3**	**5.3 ± 3.4**	**0.788**	**0.459**	**0.049**

*Note*: Values are means ± SD. Bold indicates significance *P* < 0.05. *Indicates a Condition specific Pre to Post difference (*P* < 0.05). Abbreviations: CO_2_^Abs^, absolute cerebrovascular reactivity to CO_2_; CO_2_^Rel^, relative cerebrovascular reactivity to CO_2_; MCAv, middle cerebral artery; PCAv, posterior cerebral artery; ${\dot{Q}}_{\mathrm{ICA}}$, internal carotid artery blood flow.

### 3.3 Dynamic onset response to hypercapnia

The inclusion criteria of *r* ^2^ > 0.5 was not met for the MCA velocity dynamic onset response of six participants; the PCA velocity dynamic onset response of five participants; or the ICA_Shear‐rate_ and ${\dot{Q}}_{\mathrm{ICA}}$ dynamic onset response of four participants. There was no significant main effect of time, condition or interaction for the τ, Δ_A_ or MRT of the PCA velocity, ICA_Shear‐rate_, or ${\dot{Q}}_{\mathrm{ICA}}$ (Table 5).

**TABLE 5:** Dynamic responses at the onset of hypercapnia pre and post the experimental conditions.

							*P* (ANOVA main effects)		
		*n*	Pre SIT	Post SIT	Pre CYCLE	Post CYCLE	Time	Condition	Time × Condition
MCA velocity	Δ_A_ (cm s^−1^)	13	29.8 ± 11.9	28.6 ± 11.2	27.4 ± 13.4	26.7 ± 11.4	0.720	0.583	0.926
	τ (s)	13	**57.2 ± 32.6**	76.7 ± 55.2*	**64.1 ± 40.4**	**52.3 ± 28.8**	**0.547**	**0.529**	**0.050**
	MRT (s)	13	60.1 ± 31.1	77.8 ± 55.2	77.0 ± 47.0	63.4 ± 42.5	0.784	0.934	0.100
PCA velocity	Δ_A_ (cm s^−1^)	8	15.1 ± 6.5	13.7 ± 9.8	11.7 ± 6.2	13.6 ± 5.5	0.495	0.612	0.227
	τ (s)	8	51.3 ± 34.7	34.7 ± 26.5	40.2 ± 34.0	21.3 ± 8.6	0.439	0.214	0.906
	MRT (s)	8	54.0 ± 32.0	35.5 ± 25.8	47.4 ± 35.7	25.2 ± 9.6	0.296	0.386	0.855
ICA_Shear‐rate_	Δ_A_ (s^−1^)	8	176.1 ± 71.3	137.8 ± 35.3	206.6 ± 91.0	181.2 ± 57.2	0.986	0.075	0.812
	τ (s)	8	79.8 ± 44.7	34.2 ± 23.1	63.1 ± 50.7	44.9 ± 31.2	0.371	0.372	0.828
	MRT (s)	8	90.9 ± 53.1	40.8 ± 23.0	85.4 ± 59.5	61.5 ± 43.5	0.243	0.673	0.437
${\dot{Q}}_{\mathrm{ICA}}$	Δ_A_ (ml min^−1^)	8	116.5 ± 40.4	111.9 ± 38.8	132.5 ± 46.4	123.4 ± 55.5	0.945	0.402	0.900
	τ (s)	8	68.1 ± 57.6	64.1 ± 40.9	71.1 ± 48.6	48.6 ± 31.0	0.895	0.683	0.602
	MRT (s)	8	72.1 ± 56.4	69.1 ± 39.9	88.3 ± 64.7	82.0 ± 56.0	0.851	0.424	0.938

*Note*: Values are means ± SD. Bold indicates significance *P* < 0.05. *Indicates a Condition specific Pre to Post difference (*P* < 0.05). Abbreviations: Δ_A_, amplitude; ICA, internal carotid artery; MCA, middle cerebral artery; MRT, mean response time; PCA, posterior cerebral artery; ${\dot{Q}}_{\mathrm{ICA}}$, internal carotid artery blood flow.

**FIGURE 4:**
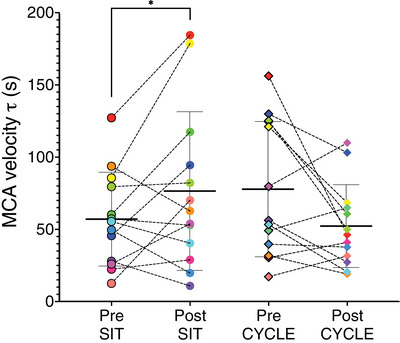
Middle cerebral artery tau Pre and Post experimental conditions. Circles represent the SIT condition and diamonds represent the CYCLE condition. Middle cerebral artery (MCA) velocity tau (τ) of each participant is represented by a single colour at all assessments. *P⩽0.05.

A significant time by condition interaction was present for MCA velocity τ (*P* = 0.05), with a slower τ following SIT (CI: −43.7 to 4.6 s, *P* = 0.05; see Figure 4).

### 3.4 Associations between habitual physical activity, sedentary behaviour, cerebrovascular reactivity to CO_2_ and kinetic onset responses to CO_2_

Average habitual physical activity for the group was 11,885 ± 3441. Average sedentary time was 470 ± 93 min per day. There were fewer children who had both habitual physical activity and sedentary behaviour data and cerebrovascular data, but steps per day and sedentary time did not differ between the whole group, the 13 with MCA data and the eight with ICA data. No correlations were found between sedentary time and any of the cerebrovascular variables. No correlations were found between steps per day, resting MCA velocity, ${\dot{Q}}_{\mathrm{ICA}}$, ICA_Shear‐rate_ or cerebrovascular reactivity to CO_2_. Significant correlations were found between steps per day and MCA velocity τ (*t* = 0.745, *P* = 0.001; *n* = 13), and steps per day and ${\dot{Q}}_{\mathrm{ICA}}$ τ (*t* = 0.541, *P* = 0.025; *n* = 8).

## 4 DISCUSSION

To our knowledge this is the first study to demonstrate that 3 h of uninterrupted sitting reduces relative cerebrovascular reactivity to CO_2_ and slows the onset response time (τ) to hypercapnia in children. Importantly, when the 3 h of sitting was broken up with a 10‐min exercise break each hour, these declines were prevented. In support of our hypothesis, 3 h of uninterrupted sitting reduced ${\dot{Q}}_{\mathrm{ICA}}$ relative cerebrovascular reactivity to CO_2_ from 5.0% to 3.3% and slowed the MCA velocity hypercapnic onset response τ by, on average, 19.5 s.

Previous work with adults has only shown a decline in MCA velocity, but not cerebrovascular reactivity to CO_2_, and it was concluded that the cerebrovasculature has a better functional capacity to resist sitting‐induced vasodilatory deficits (Carter et al., 2018). In contrast, we report reductions in ${\dot{Q}}_{\mathrm{ICA}}$ relative cerebrovascular reactivity to CO_2_. It is important to note that we do not find a reduction in absolute ${\dot{Q}}_{\mathrm{ICA}}$reactivity to CO_2_ with prolonged sitting. There is no standardized approach for the calculation of cerebrovascular reactivity to CO_2_ (Skow et al., 2013; Willie et al., 2012) and given the individual differences in baseline flow from pre to post‐hypercapnia, we calculated reactivity in both absolute and relative terms. Our disparate relative and absolute findings may simply be a reflection of these differing baseline values, which are accounted for when normalized to baseline.

The reduced ${\dot{Q}}_{\mathrm{ICA}}$ relative cerebrovascular reactivity to CO_2_ and slower MCA velocity τ may reflect reductions in cortical activity and therefore cortical perfusion and oxygen delivery with sitting (Pinto et al., 2023). Although speculative, this may account for the regional differences we report. In comparison to the more sluggish MCA velocity τ, we find no changes in the PCA with sitting. Conversely, it is possible that the posterior circulation is less impacted by sitting, given the posterior cerebral circulation supplies the brainstem where cardiovascular and respiratory control centres are located.

Impairments in cerebrovascular reactivity to CO_2_ have been linked to cognitive decline in adults (Sabayan et al., 2012; Yang et al., 2017). The young brain has a high metabolic demand that is met by a correspondingly high level of cerebral perfusion to meet this demand (Vandekar et al., 2019). As a result, it has been suggested that sitting‐induced declines in cerebrovascular reactivity to CO_2_ may predispose the child to greater cognitive maladaptation (Griffiths et al., 2021). There was no impact of sitting on any of the NVC outcomes, likely reflecting the absence of any cognitive deficits (see Griffiths et al. (2021) for a detailed review). If sitting induced declines in cerebrovascular reactivity are transitory and prevented by exercise, it is likely that there is little or no impact on cognitive function. Likewise, it should be noted that in order to maintain ecological validity and compliance of the children, participants were allowed to entertain themselves as they pleased (using personal electronic devices). If the occipital lobe was continually being stimulated throughout the sitting bouts (with and without exercise breaks), this may be enough to protect against the impact of prolonged sitting, and if this is the case, an important future question is what determines ‘good’ and ‘bad’ sitting.

Previous work in children reported increased sedentary time was associated with a decline in shear rate, suggesting cerebrovascular vasodilatory function may be impacted by prolonged sitting (Tallon et al., 2021). However, in the current study, the decline in ${\dot{Q}}_{\mathrm{ICA}}$ cerebrovascular reactivity to CO_2_ and correspondingly slower MCA velocity τ we report were not accompanied by any change in shear rate. Additionally, we show no relationship between sedentary time and ICA shear rate. It is possible that in a habitual state shear rate does decline, but in our acute experiment shear rate is not reduced. It is also worth noting that the small sample who had habitual sedentary time in the current study were active, and only three children had levels of habitual sedentary behaviour that were previously shown to be associated with a reduced ICA shear rate (Tallon et al., 2021). We do report significant positive correlations between steps per day, MCA velocity and ${\dot{Q}}_{\mathrm{ICA}}$ τ, suggesting higher levels of physical activity are associated with a slower onset response; however, any conclusions should be tempered given the very small sample size.

It has been proposed that sitting induces venous pooling in the lower limb, which in turn reduces stroke volume and may account for declines in cerebrovascular function (Hachiya et al., 2010); however, the surrogate measure of blood pooling used, calf circumference, was unchanged during the intervention. Likewise other cardiovascular measures (MAP and HR) were unchanged. There is evidence that prolonged sitting increases insulin concentration and glucose (Loh et al., 2020) and the ensuing hyperglycaemia alters cerebral glucose utilization kinetics (Wheeler et al., 2017). In response to hyperglycaemia, there are reductions in regional cerebral blood flow and increases in insulin that facilitate glucose clearance and may result in the sitting‐induced reductions in cerebral reactivity that we report (Wheeler et al., 2017). These same cardiometabolic parameters are positively impacted by exercise and potentially may contribute to the preservation of cerebrovascular function when sitting is broken up with exercise breaks noted by us and others (Carter et al., 2018; Wheeler et al., 2019). Exercise is clearly beneficial, but determining the optimal dose of exercise for preventing sitting‐induced declines in the cerebrovasculature will be essential for informing future guidelines and interventions.

The participants of this present study were otherwise healthy, normal‐weight children, and how these findings extend to other groups will require further investigation. Hypertensive, obese children have a reduced cerebrovascular reactivity to CO_2_, compared to healthy age and sex‐matched controls, which has been associated with poorer executive function (Kupferman et al., 2013). In addition, children who are obese experience significantly longer sedentary bouts and fewer active breaks compared to children of a healthy weight (Colley et al., 2013; McManus et al., 2011). Whether hypertensive, obese children would experience greater detriment to cerebrovascular function following an acute bout of prolonged sitting, and whether this could be mitigated when prolonged sitting is interrupted is unknown. Furthermore, while we controlled for maturation as a covariate within analyses, the population within this study comprised mostly pre‐PHV participants. Additionally, data loss resulted in a predominately male sample, which prevented exploration of sex differences. Whether the impact of sitting varies throughout growth and development in children and adolescents and whether there are differences with maturation and sex will be important to establish.

### 4.1 Limitations

This study is not without limitations. It was a demanding and time‐consuming project for the children and the families involved, comprising three visits totalling approximately 15 h in the laboratory. As expected, there was a loss of participants from intake through to completion of the study; however, despite the intensive protocol, the attrition rate is similar to other groups who have completed randomized crossover trials in this population (Belcher et al., 2015). That said, data loss was considerable, particularly for the kinetic onset response where only 13 of the 31 who completed the trial had MCA data and only eight had PCA or ICA onset response data. It will be important to establish whether the overall findings of this study are reproducible to understand the impact of the small sample. To maintain compliance, make involvement enjoyable and maintain the ecological validity of the protocol, the participants were allowed to use personal electronic devices while sitting with or without exercise breaks, which likely impacted the NVC measure. Additionally, the use of fixed concentration of hypercapnia has some limitations with regard to individual variation in the change in $P_{\mathrm{ETC}O_{2}}$, and the inability to hold $P_{\mathrm{ET}O_{2}}$ constant (see Tallon et al. (2020) for a detailed discussion). We feel, however, this would have a trivial influence, if any, and not alter our results.

### 4.2 Conclusion

This is the first study to investigate the impact of an acute bout of prolonged sitting on cerebrovascular function of children. While there was no significant decrease in NVC, the decreased ${\dot{Q}}_{\mathrm{ICA}}$ cerebrovascular reactivity to CO_2_ and slowed MCA velocity dynamic onset response following an acute bout of uninterrupted sitting suggest excessive sedentary behaviour does impact cerebrovascular function in childhood. Importantly, however, intermittent exercise breaks prevented this impairment. Studies incorporating cerebrovascular function, cognitive function, as well as the moderating and mediating effects of the cardiometabolic changes that accompany sitting are needed to determine the significance of the changes noted with uninterrupted sitting on cerebrovascular function.

## AUTHOR CONTRIBUTIONS

McManus, Green, Tremblay, Ainslie and Tallon conceived and designed the research. Tallon, Nowak‐Flück, Rieger and McManus assisted with data collection. Tallon and McManus analysed data. Tallon, Rieger, Green, Tremblay, Ainslie and McManus interpreted results. Tallon prepared figures. Tallon and McManus drafted manuscript. Tallon, Nowak‐Flück, Rieger Green, Tremblay, Ainslie and McManus edited and revised the manuscript. All authors have read and approved the final version of this manuscript and agree to be accountable for all aspects of the work in ensuring that questions related to the accuracy or integrity of any part of the work are appropriately investigated and resolved. All persons designated as authors qualify for authorship, and all those who qualify for authorship are listed.

## CONFLICT OF INTEREST

The authors have no competing interests, financial or otherwise, to declare.

## Data Availability

The data that support the findings of the present study are available from the corresponding author upon reasonable request.
